# Social and Environmental Influences on Physical Activity Behaviours

**DOI:** 10.3390/ijerph15010169

**Published:** 2018-01-22

**Authors:** James Dollman

**Affiliations:** Alliance for Research in Exercise, Nutrition and Activity, University of South Australia, Adelaide 5000, Australia; james.dollman@unisa.edu.au; Tel.: +61-8-83021413

Physical activity promotion has met with limited success across a range of demographic indicators, largely due to our poor understanding of how drivers of physical activity behaviours vary by context and setting [1]. Despite the heterogeneity in the literature with regard to study populations and methodological approaches to measuring physical activity, there is now widespread support for a social ecological framework for directing our research efforts. This model acknowledges the roles of the social, physical and policy environments, and the interactions between these and psychological attributes, in shaping physical activity levels across a diverse range of demographic groups. Accordingly, this Special Issue of International Journal of Environmental Research and Public Health invited contributions that provide recent evidence for associations of physical activity, either in a ‘global’ sense or in a specific context, such as organised sport, with salient social and/or physical environmental predictors of these behaviours. Consequently, we present 17 high quality articles that collectively span: adults and adolescents; males and females; social (including pets!) and built environmental predictors; qualitative and quantitative data; and objectively measured and self-reported physical activity. Some studies took a broader social determinants perspective by addressing the roles of urban versus rural residence or socioeconomic status (SES) on physical activity behaviours. Contributions came from 11 countries in total, with the majority of studies emanating from The Netherlands (3), Australia (3), Israel (2), Canada (2) and USA (2).

Nine articles focused on physical activity among children and adolescents. Best and colleagues addressed the methodological issue of measurement heterogeneity by exploring predictors of children’s physical activity that emerge regardless of whether objective or self-reported physical activity data are considered. Enjoyment, parent support and availability of sport facilities emerged as consistent predictors, suggesting that these factors should be prioritized as intervention and policy targets. The challenges of integrating physical activity options into school curriculum were addressed in a qualitative study reported by van den Berg and colleagues who interviewed primary school teachers and principals in The Netherlands. The battle for curriculum time, also reported elsewhere [2] was evident, with participants expressing support as long as the programmed physical activity benefited learning, minimally impacted curriculum time and was easy for teachers to implement. The authors concluded that persistence with this strategy is critical due to the potential to reach almost all young people in the school setting. Moran, Eizenberg and Plaut reported the relationship between walking to school and the development of navigation skills and knowledge of the neighbourhood among Israeli schoolchildren. These findings support previous evidence [3] that active commuting between home and school is beneficial, not just in terms of the energy expended during the journey, but by increasing confidence and skills to actively commute to other neighbourhood destinations. Factors affecting children’s outdoor activity were explored in two studies, contributing to our understanding of this important context for creative and social play. Moran, Plaut and Merom highlighted the importance of well-maintained and safe neighbourhood play spaces, while also shedding light on the impact of social norms and shared values on acceptability of outdoor play. Fromel and colleagues presented an interesting analysis of the importance of meeting activity preferences among youth in Poland and Czech Republic. Notably, the more closely outdoor activity preferences were met, the more likely that young people would meet age-specific recommendations for daily physical activity. A key objective for policy makers should therefore be the provision of a wide ‘menu’ of outdoor activities from which young people can choose, in accord with Self Determination Theory (SDT) which posits that satisfaction of the basic needs for autonomy, competence and relatedness is important for establishing intrinsically motivated and sustained physical activity behaviour [4]. Notably, Berg and colleagues’ study of low SES Swedish children also resonated with SDT by highlighting the importance of social support from family and peers as well as varied options that match children’s skill levels. Finally, Roberts and colleagues presented intriguing evidence that dog ownership was associated with parental awareness of positive built environmental attributes (such as traffic speed restrictions) among families in the USA, the authors suggesting that this in turn might be supportive of children’s outdoor play through lower perceived neighbourhood risk.

The majority of studies of adult physical activity focused on aspects of the built environment, with two studies examining this from a socioeconomic perspective. Zandiah and colleagues used Geographic Positioning System technology to compare high and low SES neighborhoods in The Netherlands on components of walkability for older adults. Disparities in walking across SES gradients could be related to availability of green spaces and recreation centres. Highlighting the need to consider influences of physical activity in context, Micklesfield and colleagues reported that the influence of SES on physical activity among young South African women depended on whether they lived in urban or rural areas. In accord with this observation, Berry and colleagues found that neighbourhood walkability was directly associated with walking behaviour in urban but not rural Australian adults. The authors suggested that context-specific measures of walkability need to be developed to better understand the environmental influences that seem to be unique to rural settings. The challenges of promoting physical activity in rural areas of the USA were investigated by Lo et al., with residents’ preferences for structured and socially supportive settings discussed against a backdrop of limited resources to effect rapid change in these communities. The authors suggested that small, staged improvements to existing features were key to meeting substantial longer term targets. Costigan and colleagues identified features of public parks that should be targeted for improvement in their study of park use for recreational physical activity among Australian adults. Ease of access, provision of shade, safety and regular maintenance were strongly supported. Interestingly, the availability of recreational dancing in public parks was associated with overall physical activity among Colombian women, as reported by Sarmiento et al. All but one of the studies of built environment influences on physical activity were cross-sectional in design and therefore unable to identify the impact of changing salient environmental features on consequent physical activity behaviours. McCormack and colleagues’ elegantly designed study tracked Canadians who changed residence in the previous 12 months, and confirmed a relationship between changed neighbourhood walkability and changes in active commuting. The only study of social influences on adult physical activity came from Westgarth and colleagues who provided intriguing evidence for a ‘mutualistic’ association among dog owners and their dogs during walking. Dog walkers were strongly motivated by the sense that the dog also enjoyed the experience, much more so than other potential influences such as exercise benefits and social interactions while walking.

This collection of studies confirms that physical activity behaviours are shaped by all levels of the social ecological framework as well as interactions among these levels, resulting in a complex causal web of factors. To advance our understanding to the point that interventions targeting social and physical environmental influences on physical activity can be designed with confidence, research efforts need to continue to focus on context- and setting-specific factors that are amenable to change within demographic groups differentiated by age, gender, social disadvantage and geographic location.
